# Evidence of B Cell Clonality and Investigation Into Properties of the IgM in Patients With Schnitzler Syndrome

**DOI:** 10.3389/fimmu.2020.569006

**Published:** 2020-12-03

**Authors:** Shelly Pathak, Dorota Rowczenio, Samuel Lara-Reyna, Mark Kacar, Roger Owen, Gina Doody, Karoline Krause, Helen Lachmann, Rainer Doffinger, Darren Newton, Sinisa Savic

**Affiliations:** ^1^National Institute for Health Research–Leeds Musculoskeletal Biomedical Research Centre and Leeds Institute of Rheumatic and Musculoskeletal Medicine, Leeds, United Kingdom; ^2^National Amyloidosis Centre, University College London, London, United Kingdom; ^3^Department of Haematology, St James’s University Hospital, Leeds, United Kingdom; ^4^Division of Haematology and Immunology, Leeds Institute of Medical Research at St. James’s, University of Leeds, Leeds, United Kingdom; ^5^Department of Dermatology and Allergy, Allergie-Centrum-Charité, Charité—Universitätsmedizin Berlin, Berlin, Germany; ^6^Department of Clinical Biochemistry and Immunology, Addenbrooke’s Hospital, Cambridge, United Kingdom

**Keywords:** Schnitzler Syndrome, B cell repertoire, autoinflammatory diseases, paraprotein, IgM

## Abstract

The Schnitzler Syndrome (SchS) is an acquired, autoinflammatory condition successfully treated with IL-1 inhibition. The two main defining features of this late-onset condition are neutrophilic urticarial dermatoses (NUD) and the presence of an IgM monoclonal component. While the former aspect has been extensively studied in this disease setting, the enigmatic paraproteinaemia and its potential consequential effects within SchS, has not previously been thoroughly addressed. Previous studies analyzing clonal B cell repertoires have largely focused on autoimmune disorders such as Systemic Lupus Erythematous (SLE) and hematological malignancies such as Chronic Lymphocytic Leukaemia (CLL), where B-cell clonality is central to disease pathology. The present study uses next-generation sequencing to provide detailed insight into aspects of B cell VDJ recombination and properties of the resulting immunoglobulin chains. An overview of IgH regional dynamics in 10 SchS patients, with a particular focus on CDR3 sequences and VDJ gene usage is reported, highlighting the presence of specific B cell expansions. Protein microarray detected a substantial proportion of autoreactive IgM to nuclear target proteins, though a single universal target was not identified. Together, these genetic and functional findings impart new understanding into this rare disorder.

## Introduction

Systemic autoinflammatory disorders (SAID) are a group of conditions, principally caused by a dysregulated innate immune system, with neutrophils and monocytes being the central effector cells, and defined by the lack of adaptive immune involvement, particularly without a role for autoantibodies. The Schnitzler Syndrome (SchS) is thought to be an acquired condition with all the cardinal features of SAID, but with one further characteristic—the presence of IgM paraprotein (>98% of cases). This is a very rare disored with fewer than 300 cases reported in the literature (1). The defining features of SchS include rashes (neutrophilic urticarial dermatosis), fever, bone pain, paraproteinemia, and excellent symptomatic response to anti–IL-1 blocking therapies. The latter feature, in particular, is suggestive of shared aetiology with Cryopyrin (NLRP3)–associated periodic syndrome (CAPS or NLRP3-AID), which is an inherited or, in rare circumstances, an acquired disorder associated with gain of function mutations in *NLRP3* leading to uncontrolled release of IL-1. However, extensive studies have failed to demonstrate the presence of *NLRP3* mutations (hereditary or somatic) in SchS patients (2, 3). In the majority of SchS patients the presence of paraprotein is due to underlying monoclonal gammopathy of undetermined significance (MGUS). A small proportion of patients (up to 20%) go on to develop frank lymphoproliferative disorders (LPD) including Waldenström’s Macroglobulinaemia (WM) (1, 4) and this association suggests an alternative cause for SchS. For example, somatic gain of function mutations in adaptor protein *MYD88* (in particular p.L265P variant) have been closely linked with the aetiology of WM (5) and MyD88-mediated cellular pathways provide a biological link with IL-1 signaling. However, our recent study found that only 9/30 SchS patients carry the p.L265P *MYD88* variant (1). Furthermore, the same study found that SchS patients did not demonstrate increased incidence of clonal hemopoiesis, which has previously been linked with enhanced pyroptosis (inflammatory cell death) and NLRP3 activation (2, 6).

In addition to the lack of evidence for a genetic origin of SchS, the precise role of paraproteinaemia in this condition has not been fully investigated. Even when the underlying cause of paraprotein is MGUS, a non-malignant state frequently observed in SchS patients, the paraprotein alone can occasionally cause significant clinical consequences. A range of such clinical disorders with predominantly dermatological manifestations, for example, cryoglobulinemic vasculitis and acquired C1-inhibitor deficiency, have collectively been referred to as monoclonal gammopathy of cutaneous significance (7). In the case of Cold Agglutinin Disease (CAD), in the context of low-grade LPD for example, IgM paraprotein has uniform epitope specificity targeting red blood cells and causing their destruction (8). By definition, almost all SchS patients have a monoclonal IgM gammopathy mainly associated with a kappa light chain, suggesting that a similar autoreactive phenomenon could be involved in disease pathogenesis. A previous study indicated that IgM skin deposits played a role in the pathophysiology of the rash (8), but subsequent review of 83 skin biopsies, which included staining for immune deposits, found IgM deposition in only 23% of samples (1).

Several methods can be used to investigate the antigenic binding properties of the SchS related paraprotein. In the case of SchS where there are no prior apparent clues to the nature of self-antigen, a non-restrictive, unbiased, proteome-wide approach is a reasonable initial step. Similar protein microarray technology has been used successfully in the past to identify relevant autoantibody targets in autoimmune diseases (9). At the same time, High Throughput Sequencing (HTS) can be employed to analyze the variety of the B cell repertoire. This is achieved by analyzing the V (Variable), D (Diversity), and J (Junctional) (VDJ) segment composition of the immunoglobulin heavy chain (IgH) and by determining the amino acid sequence of the region most important for antigen binding, complementarity determining region 3 (CDR3). Preferential expansion of B cells with specific VDJ gene segments may result in a restricted immune repertoire thereby constricting diversity, as potential specificities to antigens are reduced (10). Increased usage of specific VDJ genes are known to be a feature of autoimmune diseases such as rheumatoid arthritis and systemic lupus erythematous (SLE), where a bias toward IGVH4 has been demonstrated (11, 12). With LPD’s such as WM and CLL overrepresentation of IGHV3 has been reported (13, 14). The potential value of studying B cell clonality in SchS patients is two-fold. This analysis might provide circumstantial evidence that there is a common antigenic target of the IgM paraprotein and furthermore and, diagnostically, increasing B-cell clonality might be predictive of progression to LPD.

In this study, we used the combination of protein microarray, direct target interrogation and high-throughput sequencing of the IGH regions to determine the antigen specificity of SchS related paraprotein. Our analysis demonstrate that, while SchS patients show evidence of a skewed IGH repertoire, the composition of common antigenic targets exclusive to SchS, if these indeed exist, remain elusive.

## Methods

### Ethics and Patients

Informed, written consent was obtained from the participants of this study in accordance with the Declaration of Helsinki. Genomic DNA was extracted from peripheral blood of 10 SchS patients for IGH repertoire analyses. Serum was isolated from the peripheral blood of another three SchS, two WM, and one NLRP3-AID patient for IgM isolation.

**Tables 1** and **2** show the demographic and clinical features of SchS and disease control groups used in the IgM array analysis and IGH repertoire sequencing.

**Table 1:** Patients included in the IgM isolation and protein array experiments.

Patient ID	Age of SchS symptom onset	Sex(M/F)	MYD88 L265P(Yes/No)	IgM subtype (kappa or lambda)	IgMk para-protein levels (g/l)	Isolated IgM levels (g/L)
SchS-1	43.5	F	Yes	IgMk	16.7	0.55
SchS-2	60.7	F	No	IgMk	9	0.73
SchS-3	51.0	F	Yes	IgMk	14	0.33
WM-1	N/A	M	Yes	IgMk	7.4	0.33
WM-2	N/A	F	Yes	IgMk	8	0.61
NLRP3-AID	N/A	M	N/D	N/A	1.56^*^	0.28

^*^polyclonal IgM levels.

**Table 2:** Clinical data and previous findings pertaining to the SchS patients included in the IgH repertoire sequencing work.

ID	Sex	Age at symptom onset (years)	Duration of ShcS related symptoms (years)	CRP levels (mg/dl)	Para-protein	IgMk para-protein levels (g/l)	Response to IL-1 inhibition	Bone marrow histology	Genes/Variants identified by MDS panel	*MYD88* L265P?
**4**	male	36.8	16.8	4.0	IgMk	3	Partial	No overt LPL		No
**5**	male	43.9	8.7	6.0	IgGλ	N/A	complete	15% plasma cell		No
**6**	female	44.8	17.1	12.0	IgMk	1	complete	No overt LPL		No
**7**	female	49.6	8.4	8.9	IgMk	4	complete	not done		No
**8**	male	52.8	15.3	4.5	IgMλ (IF)	N/A	complete	No overt LPL		No
**9**	male	58.1	14.4	4.9	IgMk	3	complete	LPL	*STAG-2* c.559C>T p.Gln187* Predicted: pathogenic VAF: 0.081	No
**10**	male	59.6	13.3	7.9	IgMλ	7	died before treatment	No overt LPL		No
**11**	male	61.7	13.3	Not avail	IgMk	5	complete	No overt LPL		No
**12**	female	68.4	21.3	14.0	IgMk	8	complete	No overt LPL		No
**13**	male	78.9	13.6	14.3	IgMk	7	complete	No overt LPL		L265P

IF, Immunofluorescence; VAF, Variant allele frequency; LPL, lymphoproliferative lymphoma.

*here is used as a standard way to mark the effects of mutation.

### IGH Repertoire Sequencing

Leader sequence and JH consensus primers (appendix) containing Nextera linkers were used to amplify the IGH region from 10 SchS patients’ genomic DNA, which was isolated from whole blood. A second PCR added the Nextera sequencing adaptors and indices (Illumina) onto the purified IGH amplicons. Details regarding the primers, thermocycling conditions, agarose gel electrophoresis, and gel purification are described in the supplemental methods. The concentrations of the individual libraries were determined by pico-green fluorescence using the Quant-iT™ dsDNA Assay Kit (Invitrogen, USA) with the size of the final amplicons (~700 bp) and purity determined using the Agilent 2200 TapeStation system (Agilent Technologies, UK). Sequencing was carried out by the NGS facility within the Leeds Institute of Medical Research at St James’s Hospital, UK. NGS was performed on an Illumina MiSeq sequencing instrument (Illumina, UK), with a 600 cycle (2 × 300 bp) paired-end run, according to the manufacturer’s instructions. MiSeq reporter software demultiplexed the samples with FastQ (.fastq) files as the final output.

### Bioinformatics and Analyses

The resulting.fastq files were subject to downstream processing, analysis, and visualization. The following NGS platforms are open access web-based programs and used sequentially in this work: www.usegalaxy.org; www.imgt.org; and www.bioinf-galaxian.erasmusmc.nl/argalaxy. **Supplementary Figure 1** portrays how these bioinformatics tools are utilized, starting from the Illumina MiSeq generated fastq file (Galaxy), to the generation of CSV files for use with Microsoft Excel (15), and finally to the downloadable graphs and plots generated by AR Galaxy pertaining to VDJ and CDR3 properties.

The sequencing data has been deposited to BioPoject database (http://www.ncbi.nlm.nih.gov/bioproject/664629), submission ID: SUB8177406

BioPoject ID: PRJNA664629

### IgM Isolation

IgM isolation and subsequent purification of serum samples was carried out using the ÄKTA™ Pure system (GE Healthcare, UK) at 4°C, using prepacked HiTrap™ IgM Purification HP column as the stationary phase. IgM containing fractions were pooled; protein purity was assessed by SDS-PAGE and subjected to fluorometric quantitation using Qubit (ThermoFisher Scientific, UK).

### Protein Microarray

Isolated IgM were incubated with the HuProt™ array comprised of GST tagged proteins covering 75% of the human protein coding genome (Cambridge Protein Arrays, UK). IgM (15 µg/ml) from one NLRP3-AID, two WM, and three SchS samples were applied to the array. Following incubation of IgM, anti-human IgM Alexa Fluor IgM-488 (ug/ml) and anti-GST-Dylight-650 (0.5 ug/ml) were applied to the array in order to detect IgM and the embedded proteins (serving as a positive control). A seventh slide with no IgM antibody incubated was included as the negative control. The slides were washed and imaged with the Tecan LS400 microarray scanner. Resulting data were processed and analyzed using GenePix software (Molecular Devices, USA).

For each protein on the sample array, average fluorescence signals, and standard deviations (SDs) were calculated for the duplicate spots of each protein. Average fluorescence signals obtained from the corresponding protein on the negative control array were subtracted, yielding corrected protein specific fluorescence signals. These signal values were then log2 transformed, yielding log2 (Sample-Control). Finally, z-scores were calculated for each protein on the array, to determine signal significance within the signal distribution: z = (log2 (Sample-Control) − average log2)/SD log2. Those proteins with z-scores above 2.5 were deemed as likely interactors.

## Results

### BCR Repertoire Analysis

**Figure 1:**
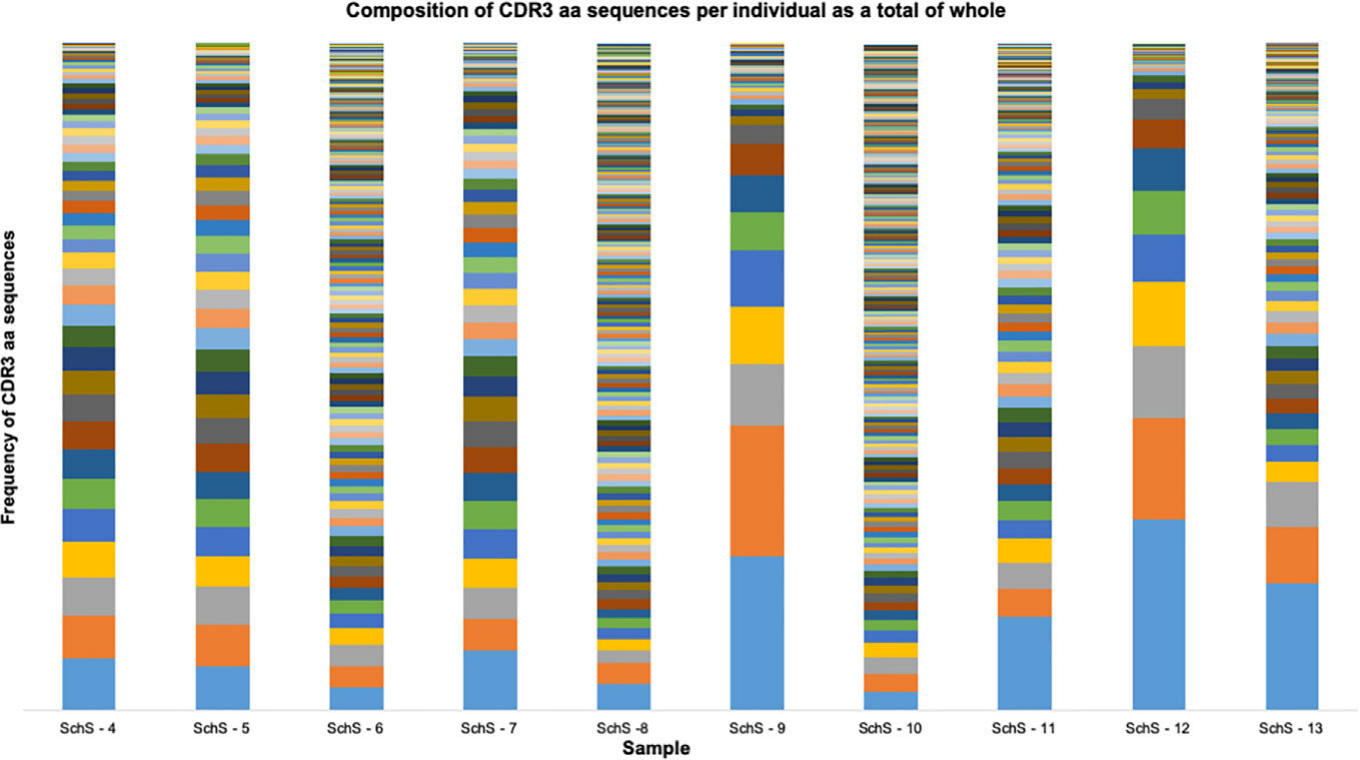
Frequency distribution of all CDR3 AA sequences identified per SchS patient (n = 10). Each bar is representative of one patient. Each color stripe is representative of a unique CDR3 found within that sample, with bar thickness pertaining to the frequency of that CDR3 AA sequence.

We used HTS of the IGH region of 10 SchS patients’ genomic DNA, in order to address the hypothesis that SchS patients exhibit a biased IGH repertoire. The IMGT numbering system was used for CDR3 identification and annotation (16). In this work, a clone is defined as a unique CDR3 sequence appearing in more than 2% of the total CDR3 sequences per individual based on previous cut-off values described by Tak et al. (>2.5%–5%) (17). Given the potential diversity of B-cell receptors generated by VDJ recombination (17), the rare probability of converging upon the same rearranged IGH sequence renders each CDR3 amino acid (AA) sequence as a unique tag for a B-cell clone (18). From the data shown in **Figure 1**, each SchS sample shows increased usage of specific CDR3 AA rearrangements above the 2% limit for clonality, though to varying degrees. SchS-9 and SchS-12 show particular skewing of the IGH repertoire indicating a highly oligoclonal rearrangements. Imperatively, no public CDR3 sequences were found in this dataset and therefore the AA sequences are unique to each participant (**Supplementary Table 1**).

### CDR3 Analysis

**Figure 2:**
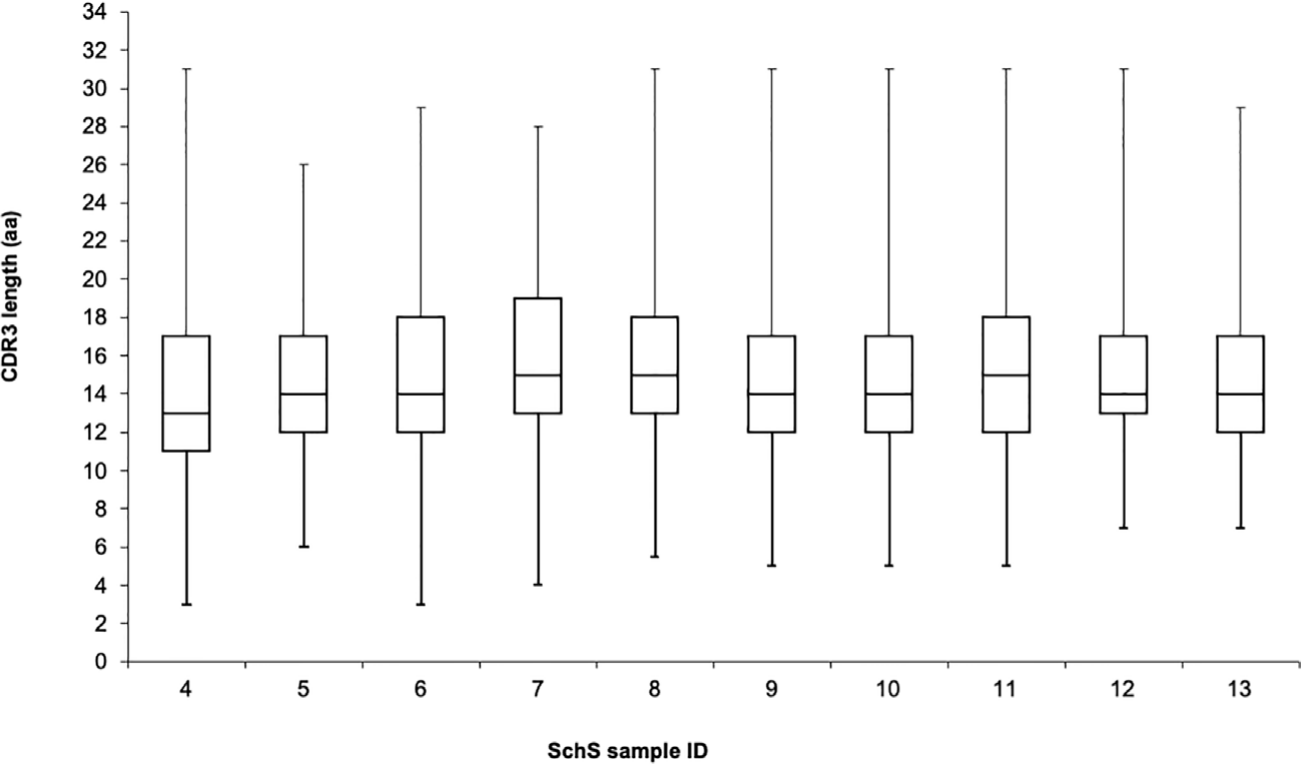
The CDR3 length data comparison obtained from the 10 SchS patients included in this work. The whiskers denote the ranges, with the interquartile and median values indicated by the boxes.

The length of the CDR region varied from 9 AA (sample 5) to 24 AA (sample 7), with the median value being 15 AA, comparable to healthy individuals (**Supplementary Table 1**). The median CDR3 lengths across the individual 10 SchS patients was either 13, 14, or 15 AA, with range between 3 and 31 AA (**Figure 2**). A large amount of published data demonstrates that the majority of “healthy” CDR3 lengths are between 8 to 18 AA with median values of 15 AA to 16 AA (19, 20), one to two AA longer than the median values found within SchS patients (**Figure 2**). The grand average of hydropathicity index (GRAVY) value represents the hydrophobicity of a given peptide. This formula calculates the sum of the hydropathy values of each AA in a sequence divided by the sequence length (based on the values provided by Kyte and Doolittle) (21). Positive GRAVY scores indicate the hydrophobic nature of the peptide, whereas negative scores denote hydrophilicity. Though the values obtained for the clones in this SchS data set were in the range of −1.54 and +0.66, 81% demonstrated a negative score indicating that the clones seen in this set of SchS are largely hydrophilic (data not shown).

**Figure 3:**
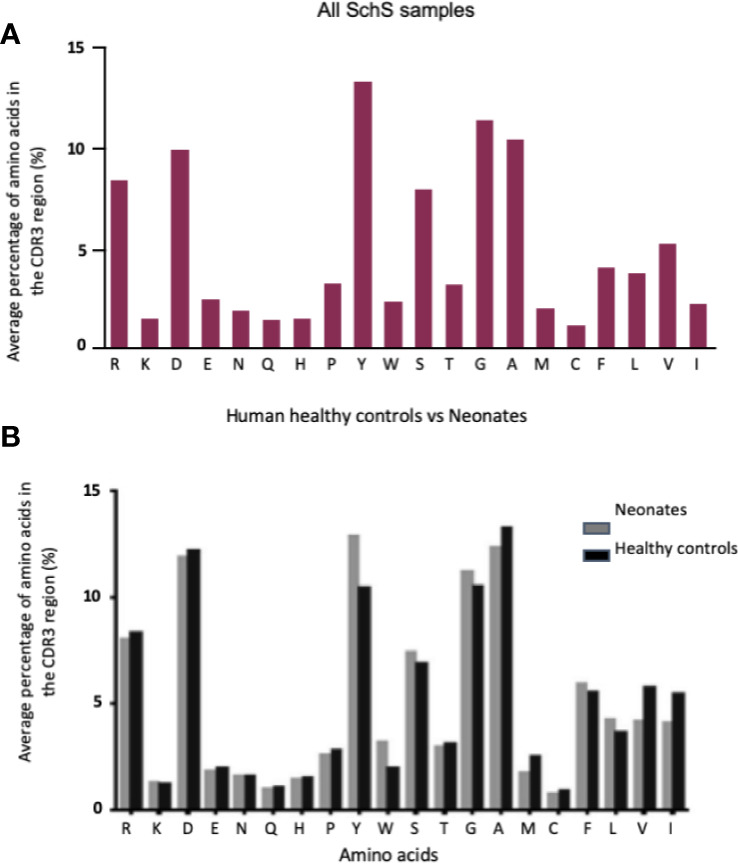
**(A, B)** Comparison of 10 SchS CDR3 AA composition **(A)** with the compositions of 2 sets of data derived from the literature **(B)**. **(B)** represents healthy controls (n = 33) vs. neonates (n = 10). Figure 4B obtained from Hong et al. (18).

The CDR3 AA compositions obtained for the SchS patients were largely comparable to the peripheral blood of 33 healthy controls and the cord blood of 10 newborns (19) (**Figures 3A, B**). Both SchS patients and healthy controls frequently use, arginine, aspartic acid, tyrosine, glycine and alanine within their CDR3 regions.

### VDJ Analysis

Within the clonal repertoire (**Supplementary Table 1**), 78% of the CDR3 AA sequences utilized the IGHV3 family, with IGHJ4 and IGHJ6 being the most common J families among these sequences. IGHD1 and IGHD6 were the most utilized D families.

**Figure 4:**
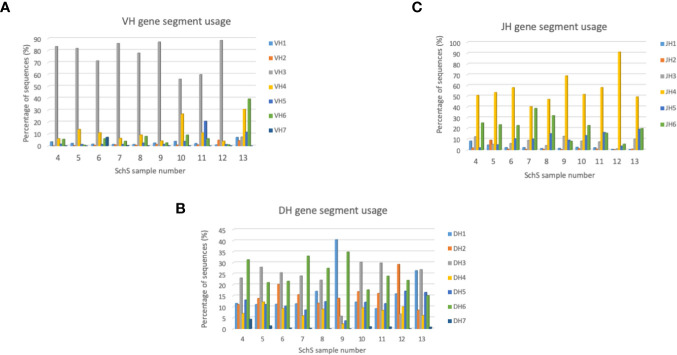
**(A–C)** Bar charts summarizing VH, DH, and JH gene usage in the SchS cohort. The percentage of sequences are on the Y axis with the sample number on the X axis. VH, DH, and JH usages are shown as the percentage of sequences on the Y axis.

Among the entire repertoire, IGHV3 genes are the most commonly employed segments (55%–88%) in nine out of the 10 SchS patients surveyed, whereas patient 13 more commonly used IGHV4 and IGHV6 (30% and 40%, respectively). IGHD3 and IGHD6 were the most frequently used gene families in seven out of the 10 samples (4–8, 11, 22), with a 25% usage each. Patient 9 preferentially employed IGHD1 at 40%, patient 12 using IGHD2 at 29% and patient 13 used both IGHD1 and IGHD3 at 26% and 25%, respectively. Each of the 10 SchS patients followed the same IGHJ usage patterns in their repertoires with IGHJ4 being the most frequent J gene used within the range of 40% to 90% (**Figure 4**).

**Figure 5:**
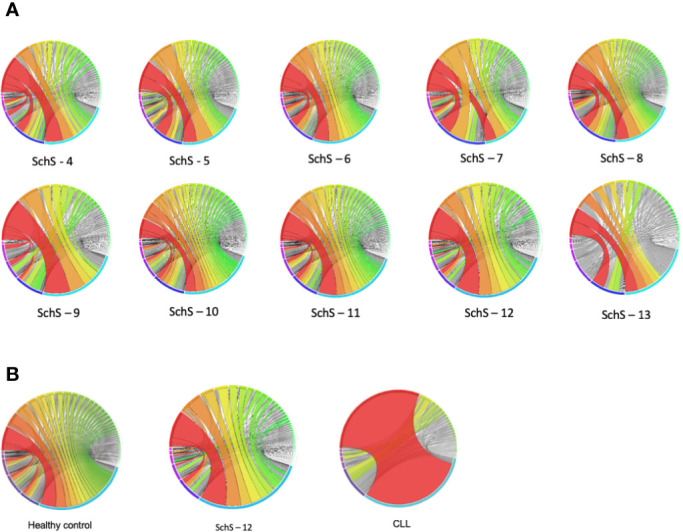
**(A, B) (A)** Circos plots demonstrating the frequency at which a V-J combination is used. The colors indicate those V-J combinations pertaining to more than 1% of the total nucleotide sequences observed, whereas the gray lines correspond to combinations constituting less than 1%. The greater the width of the ribbon, the higher the frequency of that particular VH gene combined with a specific JH gene **(B)**. comparison of representative HC and CLL circos plots with SchS.

The plots shown in **Figure 5** are a visual representation of the IGHV-IGHJ rearrangements and clearly denote overrepresentation of specific pairings. Although the prevalence of certain V-J combinations is not entirely indicative of clonality (i.e., clonal populations of B cells), preferential usage of particular gene segments points toward a skewed B cell repertoire. The light blue and navy blue arcs located within the bottom half of the circos plots denote the IGHJ4 and IGHJ6 families respectively (in sample 13, the navy blue arc represents the prevalent IGHJ5). The IGHV family arcs, within the top half of the plots are in clockwise order of most employed IGHV segment to the least. For each sample, the red and orange ribbons demonstrate the most frequent recombination within each patient. Samples 4, 5, and 7 preferentially pair the IGHV3/IGHJ4 and IGHJ6 families, whereas samples 6, 8, and 10 have a preference to joining IGHV6/IGHV3 to IGHJ4 and IGHJ6. A substantial portion of the IGH repertoire in SchS 9, 11, and 12 is restricted to IGHV3/IGHJ4 pairings, and in contrast to the other nine samples, sample 13 exhibits enhanced familial pairing of IGHV6/IGHV4 to the IGHJ4 and IGHJ5 genes.

**Figure 5B** compares circos plots from a healthy control, SchS patient 12 and a CLL patient. A diverse IGH repertoire is seen in the HC, in stark contrast to the CLL sample where clear overrepresentation of IGHV4-34 to IGHJ6 is demonstrated—resulting from the clonal expansion of a single B cell. Among the 10 SchS samples, samples 9 and 12 are the most clonal in terms of the increased frequency of specific IGH CDR3 clones (**Figure 1** and **Supplementary Table 1**). Comparison sample 12 to HC and CLL illustrates a more than one disproportionate V-J gene rearrangement (for example, IGHV3-11 to IGHJ4), but not to the single clonal expansion seen in CLL and is more visually akin to the HC plot.

It is well established in lymphoma that there is a restricted range of B cell receptor stereotypes. Using the AResT algorithm we tried to identify these patterns in the 50 largest B clones in the SchS patients. Among the patients only one, patient 5, had evidence of a clone falling into one of these stereotyped subsets (CLL#14, IGHV4-4, CDR3: CARLRAGAFDYW); this clone represented only 0.25% of the total repertoire.

### A Proteome-Wide Approach for Identifying IgM Binding Targets

**Figure 6:**
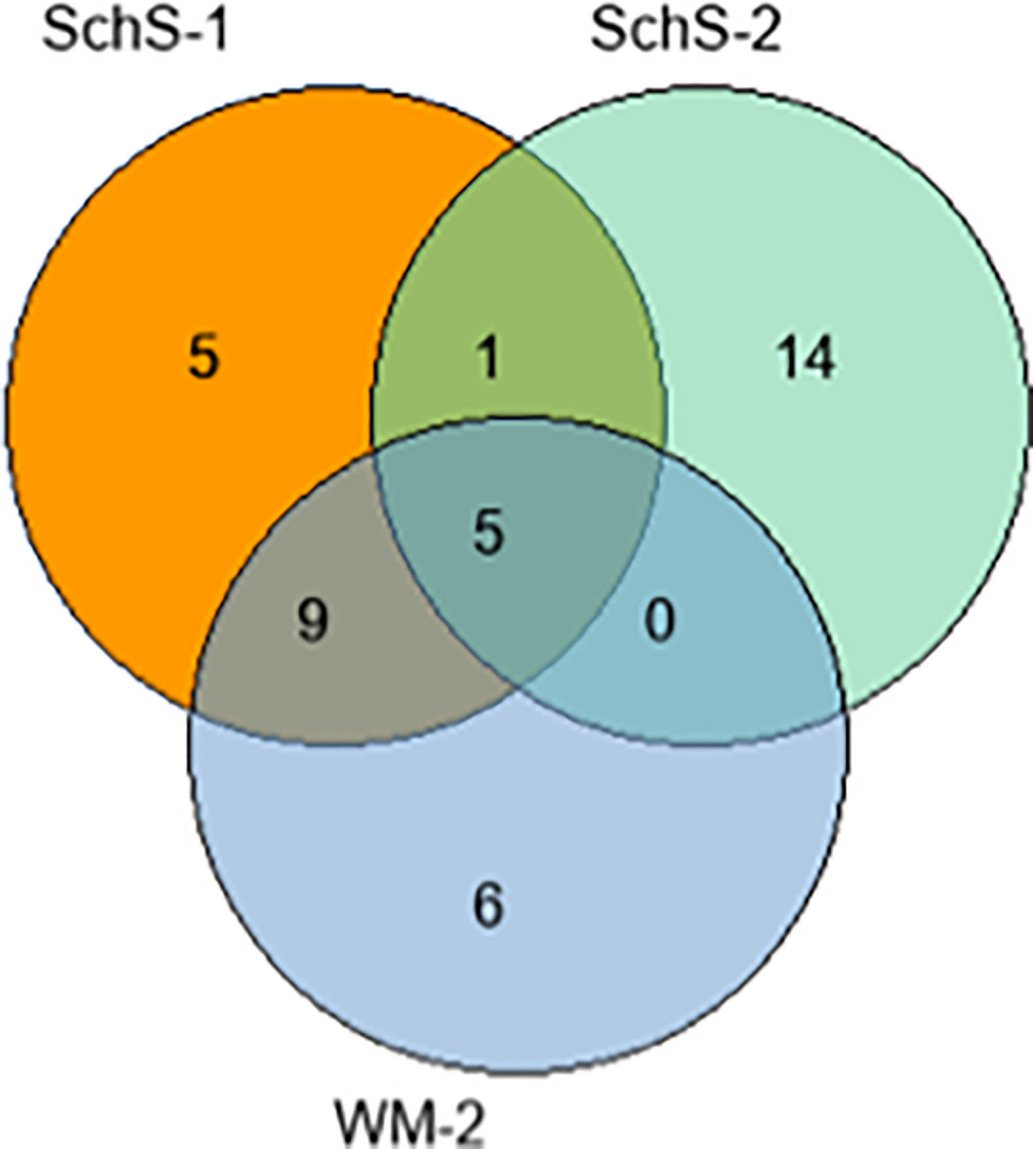
IgM targets. The top 20 hits found within samples SchS-31, NLRP3-AID-7, and WM-1 are exclusive to each patient; whereas SchS-2, SchS-23, and WM-2 share common target.

Although the BCR repertoire analysis did not demonstrate evidence of shared clonality among SchS patients, the skewed usage of particular VH genes may be consistent with recognition of a common antigen. To further test the hypothesis that the monoclonal IgM component binds to a specific protein or set of related proteins we employment of an unbiased and proteome-wide approach using isolated IgM as the probing sample. All patient samples were suitable for HuProt™ analysis with no background observed and showed a moderate strength of microarray spot interaction as indicated by the z-scores and i-scores, except for SchS-3 which demonstrated strong binding (For comprehensive list of protein array hits for all patients and controls, please see **Supplementary Table 2**). The initial analysis was restricted to the top twenty hits for each sample. This approach failed to identify a common target between all three SchS patients. The protein array was positive for a number of self-antigens, with two out of the three SchS patients and one WM patient sharing five common target proteins (ATF-1 (transcription factor), MLX (transcription factor), TLK-1 (chromatin assembly), DPP10 (peptidase), and SSH3 (protein phosphatase). H2AFY (transcriptional repressor) was a shared hit with both SchS-1 and SchS-2 (**Figure 6**). However, there were no common targets shared by all 3 SchS patients. Notably, autoimmune related hits were largely found in SchS patients (e.g. SRSF7, SART3, OGFR H2AFY, and COL4A5), indicating a SchS-IgM preponderance to recognizing self-proteins. Both SchS and WM patients demonstrated over 90% of their top 20 hits as nuclear, whereas NLRP3-AID-IgM was directed toward both nuclear and plasma membrane associated proteins. A significant proportion of these intracellular targets included transcription factors and transcriptional activators; regulators of chromatin and RNA related proteins. In contrast, extracellular antigens were largely absent with all samples. Having failed to identify a common protein shared by all SchS patient we then broadened the search. We included all interacting proteins which had z-scores above 2.5, since this technically still suggested meaningful interaction, but limited the analysis to extracellular or membrane bound proteins only, since physiologically these were the most plausible targets. Apart from DPP10 (Dipeptidyl peptidase 10), which now appeared in all SchS but not in the NLRP3-AID sample (1 WM samples was positive on initial analysis), no other common targets for found for all ShsS patients following this extended search.

## Discussion

Uniquely, among SAID, SchS is characterized by the presence of paraprotein. This study set out to investigate the role of the paraprotein and related clonal B cell population in the aetiology of this condition. We hypothesized that SchS derived IgM paraproteins have common self-antigenic targets, the binding of which leads to pathobiological effects, and consequent clinical manifestations of the condition. To explore this notion, we performed deep sequencing of the IGH regions from 10 SchS patients to determine if there is evidence of shared B cell clonality, which would imply that the manifesting paraproteinaemia may bear the same antigenic property. Furthermore, we interrogated the binding properties of the IgM isolated from three SchS patients. We used two disease controls, WM since these patients have IgM paraprotein, but no overt inflammatory complications, and NLRP3-AID, a SAID that clinically resembles SchS, but with no paraproteinemia.

The role of B cell clonality and antigenic repertoire regarding the aetiology of LPD and autoimmune diseases is complex. There is an increased incidence of LPD in systemic autoimmune diseases (15), which is thought in part to result from continuous stimulation by autoantigens and consequent narrowing of the B cell repertoire (23, 24). On the other hand, chronic self-antigen stimulation has been postulated in the aetiology of LPD. In case of CLL, the malignant B cell clones express very restricted BCR repertoire suggesting that antigenic pressure plays a part in selection of specific clonotypic BCR IG (25). However, a common antigen for LPD has been challenging to find. For example, in one study, WM-IgM was found to be directed at several self-antigens including polysaccharides, lipids, and DNA (26). Another study also looking into the immunological properties of the paraproteinaemia produced in both MGUS and MM also found no universal hits (27).

When considering CAD as an example of LPD associated with IgM paraproteinemia and a defined antigenic targets, previous studies have shown that deep sequencing methodologies can be used to identify particular IGH repertoire(s) associated with this phenomenon. A similar approach was taken in this study. Through analysis of the three general metrics of B cell repertoire skewing: CDR3 length distribution, CDR3 AA composition, and VH gene usage we found various degrees of B cell clonality in each individual case but we did not demonstrate shared B cell clonality among SchS patients. While both clonal and total populations demonstrated CDR3 lengths and AA compositions comparable to healthy controls (19), preferential usage of specific V, D, and J genes alongside biases in recombination of the latter segments points toward evidence of clonal B cell populations present in these patients. However, the degrees to which these clonal populations exist are different for each SchS patient. Presently, it is unclear if these differences could predict those patients who are greater risk of developing overt LPDs such as WM as documented in 20% of SchS patients.

To determine if autoantigens have a role in pathogenesis if SchS we used the protein microarray which contains around 75% of the annotated human protein coding genome with nearly 90% of the expressed proteins being at full-length. Interestingly, one self-antigen which was recognized by all SchS patients was DPP10. DPP10 is a dipeptidyl peptidase-like membrane protein expressed in multiple tissues (28). Physiologically, DPP10 is a plausible target since this protein is able to modulate type-A potassium channels (29). The potassium efflux is one of the main triggers for NLRP3 activation, and therefore hypothetically IgM paraprotein binding to DPP10 could affect the potassium flux and contribute to the activation of NLRP3. Moreover, *DPP10* polymorphisms have been linked to asthma and knockdown of DPP10 in bronchial epithelial cells altered responses to IL-1 stimulation (28, 30). However, DPP10 was also one of the targets which was positively identified by the IgM originating from a patient with WM, who we later confirmed did not have any inflammatory symptoms.

Further analysis found four common targets shared by two SchS and one WM patient, while generally, SchS patients showed more self-reactive profile compared to control NLRP3-AID patient. The vast majority of all positive interactions was with proteins that have either nuclear or cytosolic location. While the overarching function of the shared proteins could be broadly linked to cellular growth and pro-survival, their disparate functions and intracellular locations essentially indicate they do not belong to a specific pathway or collectively serve a combined purpose. Furthermore, considering that under physiological conditions, IgM cannot penetrate the cell, the relevance of these interactions is even more uncertain. Nevertheless, similar autoantibodies directed to the nuclear material, for example, are seen in SLE. The generation of these autoantibodies, in part, is triggered by dysregulated apoptosis and impaired clearance of the cellular debris (31). In the case of SchS enhanced inflammasome activation is well-documented. This, in turn, would result in increased pyroptosis and release of the cellular content thus facilitating the development of a more self-reactive IgM repertoire. However, these results can also be explained by the fact that total, rather than monoclonal only components of IgM were tested on the microarray. This could be resulted in non-specific, low-affinity interactions, which are generally more common with IgM compared to IgG.

The experimental evidence garnered in this work does not support the notion of an antigen-driven pathogenesis for SchS, though the suggestions of clonal B-cell populations underscores the role of the adaptive branch of the immune system within this autoinflammatory condition. However, the focus of our study was on sequencing B cell form peripheral blood, therefore it is possible that important B cell clones residing either in bone marrow, or secondary lymphoid organs would be missed by this approach. The development of lymphoproliferative disease occurs in 20% of patients, thus it is plausible to suggest that the evidence of B-cell clonality documented in SchS may be representative of different stages of pre-malignant evolution, co-existing with the “normal” B-cell repertoire. Such B-cell clones would further require malignant transformation—an event not occurring in all SchS patients. Whether the supposed event is genetic (i.e., mutations) or due to epigenetic defects remains to be explored. Nevertheless, close monitoring of SchS patients is imperative to detect progression to WM or other overt LPD development. Longitudinal analysis of the expanded CDR3 AA clones within these patients could be one approach to determine whether the latter are, in part, causal of LPD progression.

## Data Availability Statement

The datasets presented in this study can be found in online repositories. The names of the repository/repositories and accession number(s) can be found in the article/**Supplementary Material**.

## Ethics Statement

Informed consent was provided by all subjects, and the ethical approval for the study was obtained from Royal Free Hospital and University College Medical School Research Ethics Committee for this retrospective study (REC reference number 06/Q0501/42) and from Leeds (East) Research Ethics Committee (04/Q1206/107), and it was in accordance with the Declaration of Helsinki. The patients/participants provided their written informed consent to participate in this study. Written informed consent was obtained from the individual(s) for the publication of any potentially identifiable images or data included in this article.

## Author Contributions

The listed authors made the following contributions to this manuscript: SP, SL-R, GD, RD, DN, and SS performed the research and analyzed the data. MK and RD: patient data collection. DR, KK, RO, HL, and SS contributed clinical cases. SP, DN, GD, and SS: study design and writing of initial draft. All authors contributed to the article and approved the submitted version.

## Funding

This research is supported by the National Institute for Health Research (NIHR) Leeds Biomedical Research Centre and grant from SOBI pharmaceutical. The views expressed are those of the author(s) and not necessarily those of the NHS, the NIHR, or the Department of Health. SS is supported by EU Horizon 2020 research and innovation programme (ImmunAID; grant agreement number 779295). The funder bodies were not involved in the study design, collection, analysis, interpretation of data, the writing of this article or the decision to submit it for publication.

## Conflict of Interest

The authors declare that the research was conducted in the absence of any commercial or financial relationships that could be construed as a potential conflict of interest.
