# Supplementary material for: Evidence of B Cell Clonality and Investigation Into Properties of the IgM in Patients With Schnitzler Syndrome
Source: Front Immunol. 2020 Dec 3;11:569006. doi: 10.3389/fimmu.2020.569006 (PMC7793813; doi:10.3389/fimmu.2020.569006)
Supplement: Supplementary file 1 [file DataSheet_1.docx]

**Supplementary Methods**

The Leader sequence and JH consensus primers were used to amplify the IgH region of B cells via PCR, using a minimum of 200ng of gDNA from PB. The figure below demonstrates the amplification strategy employed.


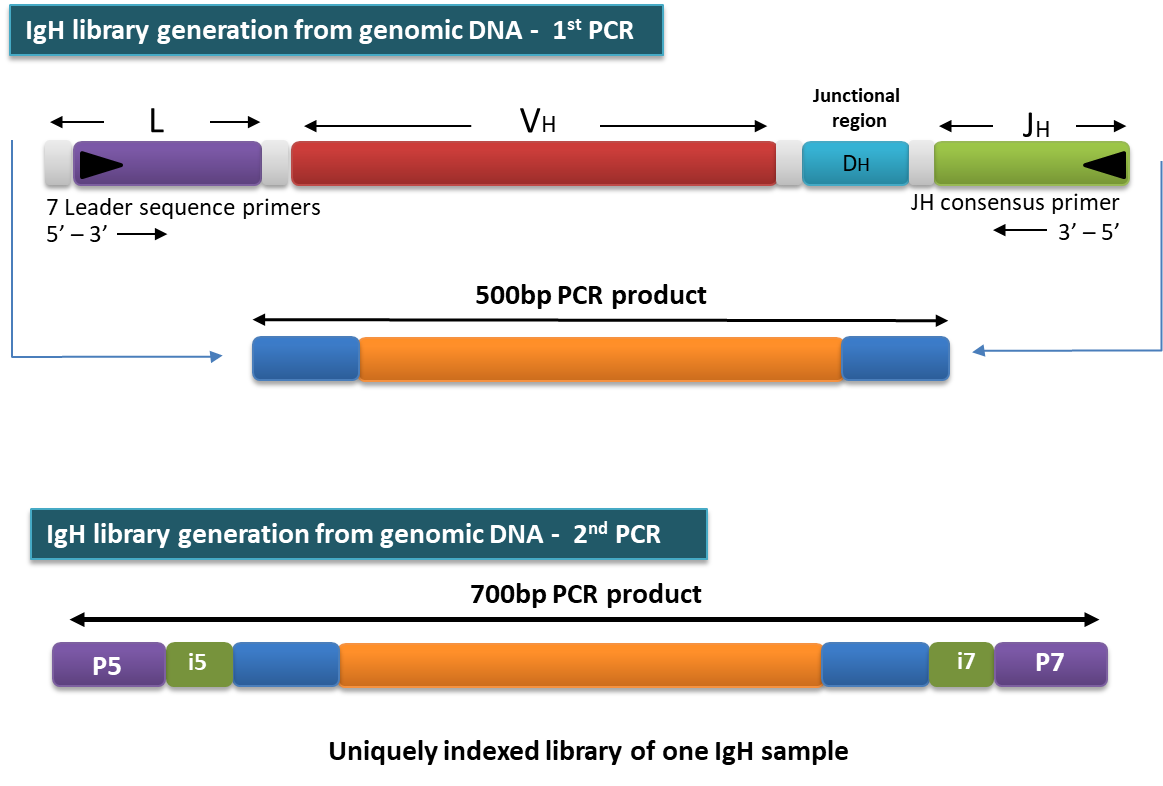


Figure 1: Amplification of the IgH region

Figure 1 is a schematic diagram of the gDNA IgH amplification from the peptide leader region across to the JH regions, with their approximate positions. The 7 leader sequence and 1 JH consensus primers are indicated with their appropriate orientations. The forward primers initiate amplification from the first, untranslated exon and are regarded as superior to the commonly used BIOMED-2 primers (1). The latter primers bind to the Framework-1 (FR-1) region of the VH segment, which is commonly subject to somatic hypermutation and thus loss of binding can occur (2). The sequence ends of the 500bp product (indicated in blue) are recognised by the Nextera indexing primers used in the second round of PCR.

| Primer name | Primer sequence 5’- 3’ |
| --- | --- |
| IGHLaa | TCGTCGGCAGCGTCAGATGTGTATAAGAGACAGAACTCACCATGGACTGSAYYTGGAG |
| IGHL2 | TCGTCCGGCAGCGTCAGATGTGTATAAGAGACAGATGGCAYACTTTGYTMCACRCTCC |
| IGHL3a | TCGTCGGCAGCGTCAGATGTGTATAAGAGACAGATGGARTTKGGGCTKWGCTGGGTTT |
| IGHL3b | TCGTCGGCAGCGTCAGATGTGTATAAGAGACAGGGCTGAGCTGGGTTTTCCTTGTTGC |
| IGHL 4 | TCGTCGGCAGCGTCAGATGTGTATAAGAGACAGCTGTGGTTCTTYCTBCTSCTGGTGG |
| IGHL 5 | TCGTCGGCAGCGTCAGATGTGTATAAGAGACAGCCTCCTCCTRGCTRTTCTCCAAG |
| IGHL 6 | TCGTCGGCAGCGTCAGATCTGTATAAGAGACAGCTGTCTCCTTCCTCATCTTCCTGCC |
| IGHJ | GTCTGTGGGCTCGGAGATGTGTATAAGAGACAGCTTACCTGAGGAGACGGTGACC |

Table 1: List of primers used to amplify the IgH locus.

Wobble bases follows the IUB code: S=C/G, Y=C/T, M=A/C, R=A/G, K=G/T, W=A/T, B=C/G/T.

##### Primary PCR

The initial PCR amplified the IgH region as illustrated in Figure 1, using 200ng of gDNA as a starting template and 10µl of Phusion Flash High-Fidelity Master Mix (Thermo-Fisher Scientific, UK), per sample. The primers, with adapter sequences recognised by the Nextera indexing primers, were diluted down 10µm working stocks to a final 1µm concentration. 0.29µl of an equimolar stock of the 7 leader primers was added to the final PCR reaction. 0.40µl from a 10µM stock of JH primer was added, with the total reaction made up to 20µl with DNase-free water. Thermocycling conditions were as follows for 35 cycles: denaturation at 98°C for 10 seconds; annealing at 65°C for 5 seconds and elongation at 72°C for 30 seconds. Following PCR amplification, 0.8µl of Midori Green Direct, (Nippon Genetics, Duren, Germany), was added to each PCR reaction to enable visualisation on the agarose gel. The gel was visualised using the blue light GelDoc system and Quantity-One analysis software (BioRad, USA). Alongside a 100bp ladder (NEB, UK), all the PCR products were run on a 2% agarose gel for 30 minutes at 90V. Bands corresponding to a region around 500bp were cut out and the DNA purified using the Zymoclean Gel DNA recovery kit (Zymo Research, USA).

##### Second round PCR

The aim of the second round PCR was to incorporate the multiplex indices and Illumina sequence tags onto the 5’ end and 3’ ends of the PCR products, in order for sample pooling and NGS. PCR with the following primers below adds the P5 and P7 termini that bind to the flowcell on the Illumina sequencer:

1. 5’ AATGATACGGCGACCACCGAGATCTACAC**[i5]**TCGTCGGCAGCGTC 3’
2. 5’ CAAGCAGAAGACGGCATACGAGAT**[i7]**GTCTCGTGGGCTCGG 3’

This step also adds the dual 8bp tags denoted by ‘i5’ and ‘i7’. The PCR mix consisted of 8µl of purified DNA from 1.2.1.2, 10µl of Phusion Flash (ThermoFisher, UK), and 1µl of each indexing primer as stated. The limited cycle PCR conditions were as follows: Initial denaturation at 95°C for 10 minutes, followed by denaturation at 95°C for 30 seconds, annealing at 60°C for 30 seconds and extension at 72°C at 45 seconds for 10 cycles. A final extension step was carried out at 72°C for 10 minutes. Agarose-gel electrophoresis, gel visualisation and purification were carried out as outlined for the primary PCR.

1. van Dongen JJ, Langerak AW, Bruggemann M, Evans PA, Hummel M, Lavender FL, et al. Design and standardization of PCR primers and protocols for detection of clonal immunoglobulin and T-cell receptor gene recombinations in suspect lymphoproliferations: report of the BIOMED-2 Concerted Action BMH4-CT98-3936. Leukemia. 2003;17(12):2257-317.

2. Agathangelidis A, Sutton LA, Hadzidimitriou A, Tresoldi C, Langerak AW, Belessi C, et al. Immunoglobulin Gene Sequence Analysis In Chronic Lymphocytic Leukemia: From Patient Material To Sequence Interpretation. J Vis Exp. 2018(141).
