# Supplementary figures and images for: Evidence of B Cell Clonality and Investigation Into Properties of the IgM in Patients With Schnitzler Syndrome
Source: Front Immunol. 2020 Dec 3;11:569006. doi: 10.3389/fimmu.2020.569006 (PMC7793813; doi:10.3389/fimmu.2020.569006)

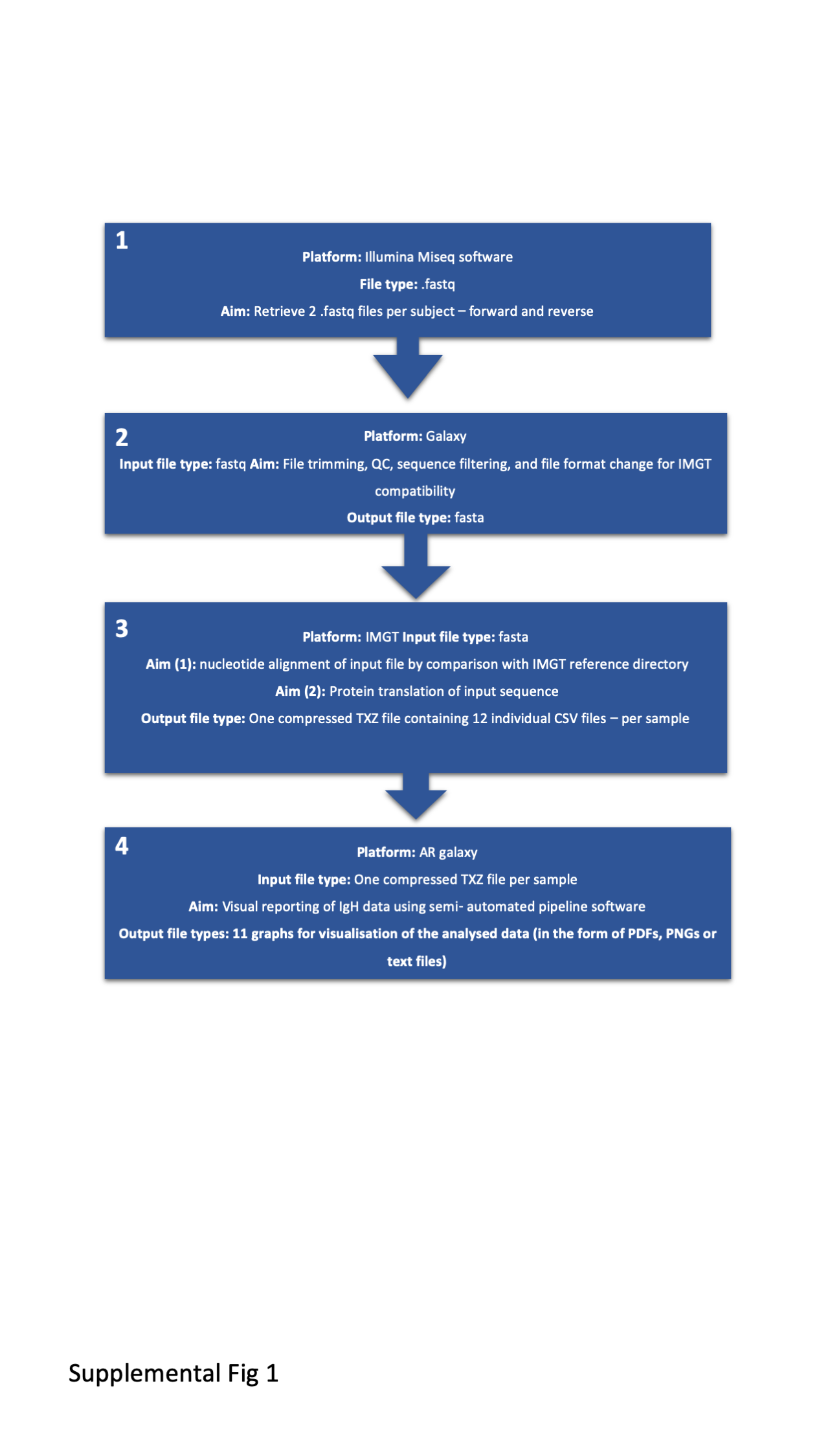

Supplement: Supplementary file 2 [file Image_1.tiff]
