# Supplementary material for: Evidence of B Cell Clonality and Investigation Into Properties of the IgM in Patients With Schnitzler Syndrome
Source: Front Immunol. 2020 Dec 3;11:569006. doi: 10.3389/fimmu.2020.569006 (PMC7793813; doi:10.3389/fimmu.2020.569006)
Supplement: Supplementary file 3 [file Table_1.docx]

Supplementary Table 1 CDR3 clones derived from the IGH sequencing data

| Sample ID | CDR3 clone sequence | % of total repertoire | VH family | DH family | JH family | V-region germline sequence identity (%) | Length (aa) | GRAVY score |
| --- | --- | --- | --- | --- | --- | --- | --- | --- |
| 4 | CAREVVGGYYGMDVW | 8 | VH3 74 | DH3 16 | JH6 | 100 | 15 | 0.17 |
|  | CARDHGGYFDYW | 6 | VH3 53 | DH4 23 | JH4 | 99 | 12 | -0.99 |
|  | CTRPNYYYYMDVW | 6 | VH3 73 |  | JH6 | 100 | 13 | -0.86 |
|  | CARDGGGYNYFDYW | 5 | VH3 11 | DH2 15 | JH4 | 100 | 14 | -0.99 |
|  | CARAVAGTTDYW | 5 | VH3 43 | DH6 19 | JH4 | 99 | 12 | 0.00 |
|  | CATQLEHDAFDIW | 5 | VH3 66 | DH1 1 | JH3 | 100 | 13 | -0.12 |
|  | CAREATGGYSYGPGGYW | 4 | VH3 11 | DH5 18 | JH4 | 100 | 17 | -0.69 |
|  | CARGSEGCFDYW | 4 | VH3 64 | DH7 27 | JH4 | 99 | 12 | -0.48 |
|  | CARDRVQHQPFGNYYYYYGMDVW | 4 | VH6 1 | DH6 19 | JH6 | 100 | 23 | -0.96 |
|  | CARDQSSGWYYFDYW | 4 | VH3 20 | DH6 19 | JH4 | 100 | 15 | -1.04 |
|  | CAKVLRRDGGALAYW | 4 | VH3 30 | DH2 21 | JH4 | 100 | 15 | 0.02 |
|  | CASPDSSGYYYW | 3 | VH3 74 | DH3 22 | JH4 | 100 | 12 | -0.70 |
|  | CARDRQQLTYW | 3 | VH3 11 | DH6 13 | JH4 | 100 | 11 | -1.30 |
|  | CARDSSGFYYYYYGMDVW | 3 | VH3 74 | DH6 19 | JH6 | 100 | 18 | -0.45 |
|  | CARVEMSVAAEDYW | 3 | VH3 73 | DH2 15 | JH4 | 96 | 14 | 0.02 |
|  | CARDFDRWAPSEYFQHW | 2 | VH3 11 | DH3 9 | JH4 | 98 | 17 | -1.12 |
|  | CASPRAFDYW | 2 | VH3 48 | DH1 26 | JH4 | 99 | 10 | -0.37 |
|  |  |  |  |  |  |  |  |  |
| 5 | CARLDRPGIAVAGQDQW | 7 | VH3 11 | DH6 19 | JH4 | 95 | 17 | -0.35 |
|  | CARDFYGDYDYW | 6 | VH3 11 | DH3 31 | JH4 | 98 | 12 | -1.09 |
|  | CAKSRRDPQLDYW | 6 | VH3 30 | DH5 24 | JH4 | 100 | 13 | -1.53 |
|  | CARDSTTVTYYYYYGMDVW | 4 | VH3 11 | DH4 11 | JH6 | 100 | 19 | -0.40 |
|  | CARGGGSYLHYYYYYGMDVW | 4 | VH3 33 | DH1 26 | JH6 | 95 | 20 | -0.41 |
|  | CAGTTVTTSYW | 4 | VH3 30 | DH4 17 | JH4 | 95 | 11 | -0.21 |
|  | CARRYFDLW | 4 | VH3 74 |  | JH2 | 100 | 9 | -0.42 |
|  | CAKGRGYGSGSSLDYW | 4 | VH3 30 | DH3 10 | JH4 | 100 | 16 | -0.71 |
|  | CARGGAAASDPFDYW | 4 | VH3 11 | DH6 13 | JH4 | 99 | 15 | -0.29 |
|  | CARVQQWLVSGYFDLW | 4 | VH3 20 | DH6 19 | JH2 | 94 | 16 | -0.24 |
|  | CARDRCSGGSCLFYYYGMDVW | 3 | VH3 30 | DH2 15 | JH6 | 96 | 21 | -0.07 |
|  | CARDWGTSSWRWFDSW | 3 | VH3 11 | DH6 13 | JH5 | 100 | 16 | -1.00 |
|  | CASIQDCSGGRCRDYW | 3 | VH3 48 | DH2 15 | JH4 | 100 | 16 | -0.64 |
|  | CARERATTVVTPAFDYW | 3 | VH3 30 | DH4 23 | JH4 | 100 | 17 | -0.16 |
|  | CAKDVLDITVAGTVDYW | 3 | VH3 23 | DH6 19 | JH4 | 92 | 17 | 0.50 |
|  | CARDSIYSSGWGMFDYW | 3 | VH3 11 | DH6 19 | JH4 | 100 | 17 | -0.33 |
|  | CARDTPYYDFWSGYGGFYGMDVW | 3 | VH3 53 | DH3 3 | JH6 | 100 | 23 | -0.47 |
|  | CARECGYSYGCFDYW | 3 | VH3 30 | DH5 18 | JH4 | 100 | 15 | -0.39 |
|  | CARDRGALNDFDIW | 2 | VH3 74 | DH5 18 | JH4 | 93 | 14 | -0.51 |
|  | CARVRGGCIRGGCHSDYW | 2 | VH3 30 | DH3 10 | JH4 | 81 | 18 | -0.38 |
|  | CARATLGSDAFDIW | 2 | VH3 74 | DH3 16 | JH3 | 100 | 14 | 0.34 |
|  | CAKDQTYYDFGLVDYW | 2 | VH3 48 | DH3 3 | JH4 | 96 | 16 | -0.54 |
|  |  |  |  |  |  |  |  |  |
| 6 | CARETAGTGAFDYW | 3 | VH6 1 | DH6 13 | JH4 | 99 | 14 | -0.37 |
|  | CARARLAYCGGDCHYYFDYW | 3 | VH3 11 | DH2 21 | JH4 | 98 | 20 | -0.33 |
|  | CARGFGAYLLDYW | 3 | VH6 1 | DH3 10 | JH4 | 99 | 13 | 0.32 |
|  | CARAADYYMDVW | 2 | VH3 30 | DH4 17 | JH6 | 99 | 12 | -0.08 |
|  | CAKAGETYGLGWYYYDSW | 2 | VH3 30 | DH5 18 | JH4 | 84 | 18 | -0.59 |
|  |  |  |  |  |  |  |  |  |
| 7 | CARASALLARALDYW | 9 | VH3 74 | DH1 14 | JH4 | 97 | 15 | 0.49 |
|  | CAKPGYNSGWYPGYYYAMDVW | 5 | VH3 11 | DH6 19 | JH6 | 98 | 21 | -0.58 |
|  | CARENWNDPLEDYW | 5 | VH3 11 | DH1 1 | JH4 | 96 | 14 | -1.58 |
|  | CAKGHAFEIW | 4 | VH6 1 | DH5 12 | JH3 | 98 | 10 | 0.15 |
|  | CARAGDIVVVPAARDYYYYGMDVW | 4 | VH3 30 | DH2 2 | JH6 | 100 | 24 | 0.20 |
|  | CANQLEGHNYGLDVW | 4 | VH3 11 | DH5 24 | JH6 | 92 | 15 | -0.51 |
|  | CARVEGYSSSWTIYYYYYGMDVW | 4 | VH3 74 | DH6 13 | JH6 | 98 | 23 | -0.26 |
|  | CARDFGGIAAAGRFGMDVW | 4 | VH3 30 | DH6 13 | JH6 | 100 | 19 | 0.39 |
|  | CASFRAVMGSPNW | 4 | VH3 74 | DH5 18 | JH5 | 100 | 13 | 0.19 |
|  | CARESSSSWPYYFDYW | 4 | VH3 11 | DH6 13 | JH4 | 100 | 16 | -0.93 |
|  | CARDGLLSGYYYYYGMDVW | 3 | VH3 30 | DH3 9 | JH6 | 100 | 19 | -0.15 |
|  | CARDAKTTVTTYPHFDYW | 3 | VH3 30 | DH4 11 | JH4 | 100 | 18 | -0.74 |
|  | CGFSTRVWGIAARRLYYGMDVW | 3 | VH3 11 | DH6 6 | JH6 | 100 | 22 | 0.15 |
|  | CAKDRAAAAGYYYYGMDVW | 3 | VH3 30 | DH6 13 | JH6 | 100 | 19 | -0.25 |
|  | CATHGNANAFDIW | 2 | VH3 30 | DH4 11 | JH3 | 100 | 13 | -0.04 |
|  | CARERADFWSGYYAAGYYYGMDVW | 2 | VH3 30 | DH3 3 | JH6 | 100 | 24 | -0.47 |
|  | CARESPSAYYSGTYYYGMDVW | 2 | VH3 11 | DH1 26 | JH6 | 100 | 21 | -0.58 |
|  | CARDRYSSSWYGGDDYW | 2 | VH3 11 | DH6 13 | JH4 | 98 | 17 | -1.42 |
|  | CARDLLSSVGRFDPW | 2 | VH3 11 | DH3 10 | JH5 | 99 | 15 | -0.10 |
|  | CTRDPRLSIPSFDDW | 2 | VH3 48 | DH2 21 | JH4 | 93 | 15 | -0.82 |
|  | CVKDSSSWSHYYYYYGMDVW | 2 | VH3 64 | DH6 13 | JH6 | 100 | 20 | 0.66 |
|  |  |  |  |  |  |  |  |  |
| 8 | CARVLVRGALDYW | 4 | VH6 1 | DH3 10 | JH4 | 100 | 13 | 0.54 |
|  | CGRDGRDGGYGMDVW | 3 | VH6 1 | DH1 26 | JH6 | 98 | 15 | -1.00 |
|  |  |  |  |  |  |  |  |  |
| 9 | CATGKWNYEENYW | 23 | VH3 74 | DH1 7 | JH4 | 97 | 13 | -1.47 |
|  | CARDNGRYAVDYC | 19 | VH3 11 | DH6 19 | JH4 | 92 | 13 | -0.75 |
|  | CARGRGGWSGALDDW | 9 | VH3 11 | DH6 19 | JH4 | 96 | 15 | -0.69 |
|  | CARDKWELQTSLLPDYW | 9 | VH3 30 | DH1 26 | JH4 | 100 | 17 | -0.76 |
|  | CARHINWGWDSW | 8 | VH3 11 | DH1 26 | JH5 | 93 | 12 | -0.82 |
|  | CARAIGAYEGLDIR | 6 | VH3 11 | DH2 8 | JH3 | 85 | 14 | 0.19 |
|  | CAKDIAPATTKTTYGMDVW | 6 | VH3 43 | DH6 25 | JH6 | 97 | 19 | -0.17 |
|  | CARGSGGNSDSPLDYW | 5 | VH3 11 | DH2 15 | JH4 | 88 | 16 | -0.89 |
|  | CARGRYGGGNAYSGTDSW | 3 | VH4 30 | DH2 21 | JH4 | 92 | 18 | -0.98 |
|  |  |  |  |  |  |  |  |  |
| 10 | CARATVTLDYYYYGMDVW | 2 | VH6 1 | DH1 1 | JH6 | 100 | 18 | 0.04 |
|  | CARDRVSLAIFDYW | 2 | VH6 1 | DH2 2 | JH4 | 97 | 14 | 0.17 |
|  | CARVTFGEFDPW | 2 | VH6 2 | DH3 10 | JH5 | 100 | 12 | -0.08 |
|  |  |  |  |  |  |  |  |  |
| 11 | CARGYSSSGGTNWFDPW | 14 | VH6 1 | DH6 13 | JH5 | 100 | 17 | -0.79 |
|  | CASDVSMSESW | 4 | VH3 74 | DH1 26 | JH4 | 95 | 11 | -0.06 |
|  | CAKAGETYGLGWYYYDSW | 4 | VH3 30 | DH5-18 | JH4 | 84 | 18 | -0.59 |
|  | CARRVGLPGGGMDVW | 4 | VH6 1 | DH3 16 | JH6 | 99 | 15 | 0.12 |
|  | CTSLHSVSSVYW | 3 | VH3 74 | DH3 9 | JH4 | 99 | 12 | 0.45 |
|  | CARDRDGYSHYFDYW | 3 | VH3 30 | DH5 24 | JH4 | 100 | 15 | -1.44 |
|  | CVRDGWELPRNYYYYMDVW | 3 | VH3 64 | DH1 26 | JH6 | 97 | 19 | -0.81 |
|  | CASPPYGDYDYW | 2 | VH3 30 | DH4 17 | JH4 | 98 | 12 | -0.99 |
|  | CARDGWYSSSWYDGTLFDYW | 2 | VH3 30 | DH6 13 | JH4 | 100 | 20 | -0.73 |
|  | CARALGWMSSSWQMGYW | 2 | VH3 30 | DH6 13 | JH4 | 100 | 17 | -0.09 |
|  | CVKNSGPGWRFDPW | 2 | VH3 30 | DH5 12 | JH5 | 93 | 14 | -0.89 |
|  | CARDRASAGEYW | 2 | VH3 74 | DH6 13 | JH4 | 95 | 12 | -0.96 |
|  |  |  |  |  |  |  |  |  |
| 12 | CAREGCGGDCYDFDYW | 29 | VH3 11 | DH2 21 | JH4 | 99 | 16 | -0.69 |
|  | CARDHSGSSYGWAFW | 15 | VH3 11 | DH1 26 | JH4 | 93 | 15 | -0.57 |
|  | CVRGEYSGYDGKGNLDYW | 11 | VH3 74 | DH5 12 | JH4 | 92 | 18 | -1.06 |
|  | CARRGSIWGVSSSGPRHFFDYW | 10 | VH3 64 | DH6 6 | JH4 | 90 | 22 | -0.48 |
|  | CAREIVAAGTLSSDYW | 7 | VH3 23 | DH6 13 | JH4 | 96 | 16 | 0.25 |
|  | CAKDTLPYGDFFDYW | 7 | VH3 30 | DH4 17 | JH4 | 96 | 15 | -0.46 |
|  | CARDRPNSGYDMDYW | 7 | VH3 30 | DH5 12 | JH4 | 95 | 15 | -1.54 |
|  | CAPTHSSGWAYFDYW | 4 | VH2 5 | DH6 19 | JH4 | 99 | 15 | -0.43 |
|  | CARHGGWIPHSFYGMDIW | 3 | VH4 39 | DH4 23 | JH6 | 83 | 18 | -0.17 |
|  |  |  |  |  |  |  |  |  |
| 13 | CARAPVRANWFDPW | 19 | VH6 1 | DH1 1 | JH4 | 100 | 14 | -0.44 |
|  | CSREHSEGSAPIWGLDYW | 9 | VH6 1 | DH5 18 | JH4 | 86 | 18 | -0.75 |
|  | CARDSWFGERVLVFDIW | 8 | VH6 1 | DH3 10 | JH3 | 77 | 17 | 0.24 |
|  | CAREGRKRGYFDYW | 4 | VH3 23 | DH5 24 | JH4 | 100 | 14 | -1.54 |
|  | CARHFSGTLPDYFDYW | 3 | VH5 51 | DH1 26 | JH4 | 99 | 16 | -0.50 |
|  | CARDSSGSSWRWFDPW | 3 | VH4 31 | DH6 13 | JH5 | 100 | 16 | -1.05 |
|  | CARQLGYGMDVW | 3 | VH5 10 | DH5 24 | JH6 | 99 | 12 | -0.03 |
|  | CVREVGATWDLGHW | 2 | VH3 13 | DH1 26 | JH6 | 95 | 14 | -0.10 |
|  | CARSSGSGSYYKYGMDVW | 2 | VH4 30 | DH3 10 | JH6 | 82 | 18 | -0.59 |
|  | CARRSYGDYSDYW | 2 | VH6 1 | DH2 2 | JH4 | 96 | 13 | -1.42 |
|  | CARNKQHHYLIFDYW | 2 | VH4 30 | DH2 2 | JH4 | 100 | 15 | -0.89 |
|  | CARLFSSAWYEDFDYW | 2 | VH4 39 | DH2 2 | JH4 | 86 | 16 | -0.34 |
|  | CALSGSYLDYFDYW | 2 | VH5 51 | DH1 26 | JH4 | 99 | 14 | -0.06 |

Sample ID, CDR3 clone sequence, percentage of the total CDR3 aa repertoire these clones make up, V, D and J gene usage, V-region germline sequence identity, aa length and the GRAVY scores are included within this table.
